# The World Health Organization Global Benchmarking Tool an Instrument to Strengthen Medical Products Regulation and Promote Universal Health Coverage

**DOI:** 10.3389/fmed.2020.00457

**Published:** 2020-08-19

**Authors:** Alireza Khadem Broojerdi, Hiiti Baran Sillo, Razieh Ostad Ali Dehaghi, Mike Ward, Mohamed Refaat, Jane Parry

**Affiliations:** ^1^Regulatory Systems Strengthening Team, Regulation and Safety Unit, World Health Organization, Geneva, Switzerland; ^2^Specialist Public Health Writer, Hamilton, ON, Canada

**Keywords:** global benchmarking tool, GBT, regulatory systems strengthening, regulation, regulation of medical products, national regulatory authority, RSS, NRA

## Abstract

National regulatory authorities (NRAs) are the gatekeepers of the supply chain of medical products, and they have a mandate to ensure the quality, safety and efficacy of medicines, vaccines, blood, and blood products, medical devices, including diagnostics and traditional, or herbal medicines. However, the majority of the world's regulators are still struggling to reach a level of maturity, whereby they have a stable, well-functioning and integrated regulatory system. The World Health Organization (WHO) has developed a Global Benchmarking Tool (GBT) as part of its five-step capacity building program to assist NRAs, using the tool, they can benchmark their own strengths and areas of weakness, and then engage in a formal benchmarking process together with WHO and international experts in order to formulate an effective and workable institutional development plan. The GBT is comprehensive across the entire product life cycle and allows benchmarking to be customized to the needs of the NRA. It has evolved from decades of experience using a variety of benchmarking tools, within WHO and other stakeholder organizations. By the end of December 2019, 26 countries had undergone formal benchmarking, and a further 54 countries had used the GBT to conduct self-benchmarking exercises assisted by WHO.

## Introduction

National regulatory authorities (NRAs) are the gatekeepers of the supply chain of medical products, and they have a mandate to ensure the quality, safety and efficacy of medicines, vaccines, blood and blood products, medical devices, including diagnostics, and traditional or herbal medicines. They work within a legal framework and set of regulatory functions spanning the medical product lifecycle, from clinical trial oversight, product marketing authorization and registration, licensing establishments, regulatory inspections, testing products, post-marketing surveillance, and vigilance activities.

When a national regulatory system is independent, efficient, science based transparent, and well-managed, it supports robust and effective medical products regulation, and medicines and other health technologies entering the market are safe, efficacious, and of assured quality. This in turn protects the population from harm due to unregulated supplies, including substandard and falsified medical products, and thus fosters confidence in the health care delivery system. A well-functioning NRA creates an environment in which medical products are appropriately manufactured, stored, distributed, and dispensed. It ensures that health professionals and patients are in a position to use medical products rationally because they have the information they need to do so, and ensures promotion and advertising is fair and balanced. It supports local production of medical products, which is key to affordability, helps create a transparent, and well-organized market for pharmaceuticals and other medical products, and enables post-marketing surveillance and integrity of the supply chain. All these facets of regulation help ensure timely access to essential medicines and enable NRAs to be prepared for better response to emergencies. Moreover, at its best, a strong NRA will perform all of these functions without creating an unnecessary regulatory burden on itself and any of the stakeholders.

As the value chain for medical products is becoming increasingly globalized, a weakness in one part of the supply chain can have adverse consequences for patients thousands of miles away. In pharmaceuticals manufacturing, for example, active pharmaceutical ingredients may be sourced from multiple countries and used for medicines production in another, before being globally distributed. A secure supply chain can be an important enabler to reduce the problem of substandard and falsified medical products.

An efficient and reliable regulatory system is also a key component of the WHO prequalification program for vaccines, as the NRA assessing providing oversight of a product applying for prequalification must be operating at an acceptable level of maturity (1). Overall, effective regulatory systems are an essential component of health systems and contribute significantly to universal health coverage.

## A Lack of Mature Regulatory Agencies

However, many countries still lack this basic building block of a well-functioning health system. According to the World Health Organization (WHO) regulatory systems strengthening database, among its 194 Member States, only 50 countries (26%) have what are considered to be mature regulatory agencies (the top or second-highest level of maturity), whilst 144 countries have suboptimal regulatory systems. Just over half, 51% (99 countries) are at the lowest level of maturity, whilst 23% (45 countries) are at the second lowest level of maturity. Although not all countries were benchmarked against WHO GBT, but the maturity level status of remaining countries have been estimated based on previous assessments done by WHO using other tools or being a Stringent Regulatory Authority (SRA). In these countries, when manufacturers of medical products want to bring their products to market, they face a landscape of disparate regulations, unclear regulatory pathways, frequent delays in accessing essential medicines and limited transparency. This suppresses innovation, drives up medicine prices and opens the door for substandard and falsified medical products. It also leaves regulators ill-prepared to deal with public health emergencies, where, for example a vaccine or medicine may need to be fast-tracked through the regulation process.

In many low- and middle-income countries, regulatory systems strengthening can be extremely challenging. NRAs are often overburdened and under-staffed, with fragmented structures or insufficient legal frameworks systems which may be difficult to reform. The first step–knowing the current state of the regulatory system where the weaknesses and gaps lie and how to go about addressing them–can be a critical task.

World Health Assembly Resolution WHA67.20 on regulatory system strengthening was adopted in May 2014 (2). This Resolution emphasized the WHO mandate and requested both WHO and Member States of low- and middle-income countries to invest more in this area and to address all health products and technologies.

## The WHO Global Benchmarking Tool

WHO supports its Member States in strengthening their regulatory systems for medical products by setting norms and standards, promoting smart regulation, identifying strengths and gaps, providing specialized technical assistance, and capacity building opportunities and advising them on issues related to quality assurance of medicines for national and international markets.

In 1997, WHO began benchmarking regulatory systems as part of its regulatory system strengthening program, initially using a set of indicators designed to evaluate regulatory oversight for vaccines. This was to address the pressing challenge of how to ensure that vaccines meet the appropriate standards of quality, safety and efficacy, whether they were used domestically in the country of manufacture, or in receiving countries. Later on, WHO expanded this programme to evaluate regulatory oversight for medicines, blood and blood products and medical devices. In 2013, WHO started to unify these parallel programs and integrate various tools developed in this regard through the development of a harmonized tool, the Global Benchmarking Tool (GBT), representing the primary means by which the WHO objectively evaluates regulatory systems (3).

The creation of the GBT also came at a time when other tools were being developed and used, including one by the Pan-American Health Organization (PAHO), as well as other non-UN agencies. The concurrent development of a plethora of tools was confusing and burdensome for NRAs and individuals responsible for using them. There were overlaps between the tools, and they had similar regulatory requirements. Unification of the WHO vaccine, medicine and broader PAHO tool, and alignment with non-WHO tools underpinned the creation of the GBT (4).

The GBT enables regulatory authorities to self-evaluate their own strengths and areas for improvement; facilitates the formulation of an institutional development plan to build upon strengths and address the identified gaps and prioritizes interventions; and facilitates the monitoring of progress and achievements. It helps countries to develop strong legal foundations and political leadership to underpin a regulatory system with a clear focus on patient safety and transparency in decision-making; and to identify and develop a core set of regulatory functions to meet country and regional needs.

The GBT evolved through a process of wide and deep consultation with WHO Member States and other stakeholders, and piloting in different regulatory settings. The consultation conducted through four face-to-face meetings and many hours of virtual discussions as well as a public consultation on the draft tool to solicit comments from a wide range of Member States and stakeholders and incorporating them into the current version. The GBT was field-tested in 2018 and the first use of the current version were conducted in March and April 2019 in Ghana and El Salvador, respectively.

One of the significant additions to the latest version of the tool, GBT Rev VI, is benchmarking modules that look at the NRA's ability to tackle the problem of substandard and falsified medical products. This is covered under several modules of the tool including the national regulatory system, market surveillance, and laboratory testing, as well as regulatory inspection, and marketing authorization The latest revision of the tool also promotes other principles of Good Regulatory Practices, including legality, impartiality, consistency, proportionality, flexibility, effectiveness, efficiency, clarity, and transparency as well as the need for implementation of risk and quality management in regulatory systems, and regulatory preparedness to face emergencies (5). Several sub indicators in the tool look at the NRA capacity and preparedness for managing emergencies.

**Figure 1:**
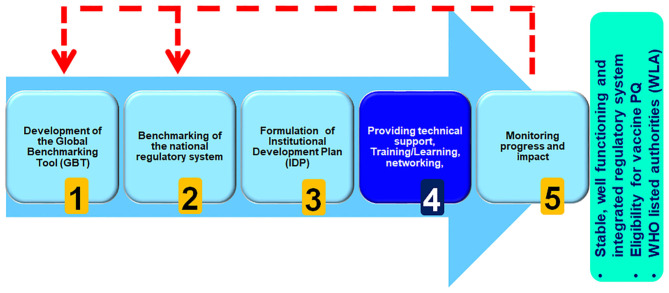
WHO five-step approach to national regulatory agency capacity building.

The WHO Regulatory Systems Strengthening programme, which aims to strengthen capacity of regional, sub-regional and national regulatory systems, works with countries to apply the GBT, as part of a five-step approach to improvement and NRA capacity building, with priority given to regulatory systems strengthening for developing countries (Figure 1).

## Structure of the Global Benchmarking Tool

The GBT is divided into four levels: (1) national regulatory system and regulatory functions; (2) indicators, (3) sub-indicators, and (4) fact sheets. In addition to the national regulatory system component that provides the framework, there are eight core regulatory functions, which between them cover the whole product life cycle of medical products (6). Core functions are agreed during the consultation process with the NRA include the following:

### Registration and Marketing Authorization

Marketing authorizations (also known as product licensing or registration) are the procedures for approval of a medical product for marketing after it has been evaluated for safety, efficacy, and quality of the product, and the appropriateness of the product information (7).

### Vigilance

Vigilance is the science and activities relating to the detection, assessment, understanding, and prevention of adverse effects or any other medical product-related problems (8).

### Market Surveillance and Control

An NRA's market surveillance and control function activities are primarily concerned with control of import activities, prevention, detection, and response to substandard and falsified medical products, quality monitoring throughout the supply chain, and control of promotional, marketing, and advertising activities (9).

### Licensing Establishments

The NRA is responsible for ensuring that all establishments throughout the medical products supply chain are licensed to undertake the respective activities (e.g., manufacturing, distribution, wholesale, retail) (10).

### Regulatory Inspection

Regulatory inspections ensure that operations are carried out in accordance with approved standards, norms, and guidelines and are in compliance with the national medical products legislation and regulations. These, in turn, should be consistent with WHO recommendations and other internationally recognized guidelines (11).

### Laboratory Testing

The laboratory testing regulatory function is intended to ensure that the National Regulatory Authority (NRA) is able to assess the quality of medical products by performing quality tests when needed (12).

### Clinical Trials Oversight

Clinical trials oversight is aimed at protecting the safety and rights of humans participating in clinical trials, ensuring that trials are adequately designed to meet scientifically sound objectives, and preventing any potential fraud, and falsification of data (13).

### National Regulatory Authority Lot Release

Lot release (also called official authority batch release) is a non-common regulatory function that does not apply to all medical products. Rather, it applies only to some specific products (e.g., vaccines) (14).

Within each regulatory function, the GBT uses a set of indicators, each with their own sub-indicators. For the sake of structural consistency across the regulatory functions and to assist benchmarking or one or more specific theme across the functions, the GBT indicators are categorized into nine categories:

1. legal provisions, regulations, and guidelines
2. organization and governance
3. policy and strategic planning
4. leadership and crisis management
5. transparency, accountability, and communication
6. quality and risk management system
7. regulatory process
8. resources (human, financial infrastructure, equipment, and information management system)
9. monitoring progress and assessing impact.

Fact sheets for each sub-indicator provide further details and clarify the scope of each sub-indicator, and there are also indicators for input, process and output. This can bring more consistency and quality to process and outcome of benchmarking. There are 268 sub-indicators in total. A four-tier scoring system measures the level of implementation and monitors the progress of each sub-indicator. The rating scale of each single sub-indicator ranges from not implemented, ongoing implementation, partially implemented, and fully implemented.

By walking through this methodical, step-wise benchmarking process, first through pre-visit of the NRA followed by self-benchmarking and subsequently through WHO formal benchmarking by an international team of experienced assessors, assembled and trained by WHO, it is possible to clearly understand the capacity of the system and the level of maturity an NRA has already reached, and from that formulate an institutional development plan to address areas for improvement and enhancing regulatory capacity of Member State.

**Figure 2:**
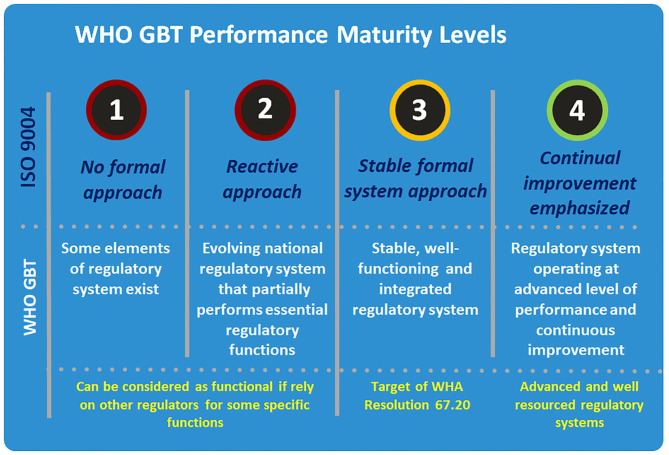
WHO global benchmarking tool performance maturity levels. Source: World health Organization.

The four performance maturity levels were adopted from the International Standard ISO 9004 (Quality management—Quality of an organization –Guidance to achieve sustained success) and are an expression of the extent to which a regulatory system has been formalized as stable, well-functioning and integrated (Figure 2). In a Level 1 NRA, some elements of regulatory system exist, whilst a Level 2 NRA will be an evolving national regulatory system that partially performs essential regulatory functions. Level 3 represents the minimum target for most NRAs to reach: a stable, well-functioning and integrated regulatory system. Level 4 NRAs exceed this required standard, and represent a regulatory system operating at advanced level of performance and continuous improvement. It is worth mentioning that the overall maturity level of a system, calculated based on the lowest maturity level of individual regulatory functions. For example, if a regulatory system is scored for all functions as ML 3 and only one function is scored as ML 2 then the overall maturity level of the regulatory system will be calculated as ML2. However, as there are many different models of regulatory systems that can fit the purpose at national level, the tool, scoring system, and methodology are adapted with some flexibility to accommodate these differences.

**Figure 3:**
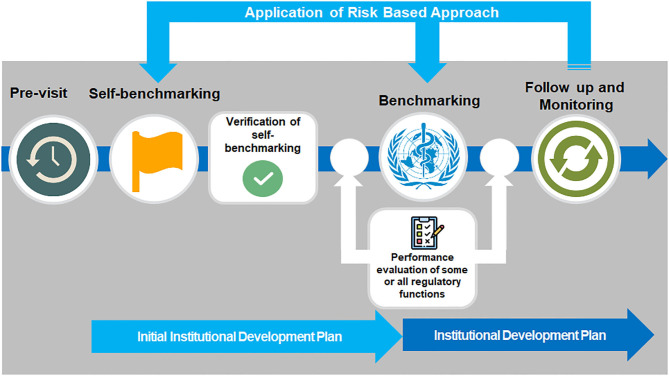
Benchmarking process.

Countries requesting assistance from WHO to benchmark their regulatory system using the GBT all follow a clear process, from planning and pre-screening, including a pre-visit mapping of the regulatory system (Figure 3). Self-benchmarking is then validated ahead of the formal benchmarking process, which can include enhanced performance evaluation of specific regulatory functions, for example observed audit for evaluation of regulatory inspection function, and vigilance field visits for evaluation of vigilance function (15).

**Figure 4:**
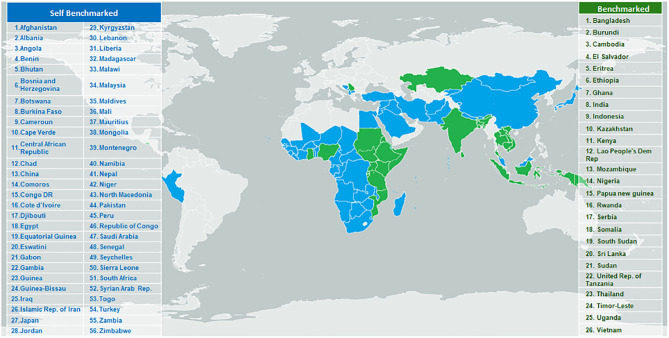
Countries/areas targeted for WHO regulatory system strengthening program and benchmarked against GBT indicators, January 2016- September 2019.

In addition to the GBT itself, WHO maintains a database of reports and IDPs of the NRAs that have been benchmarked against the GBT and this is supported by a computerized platform to facilitate the benchmarking, including the calculation of maturity levels (16). The computerized GBT (cGBT) is available upon request to Member States and organizations working with the WHO under the Coalition of Interested Parties. From January 2016 to December 2019, 26 countries underwent formal benchmarking (including Tanzania, see box 1), and 54 countries used the GBT to conduct self-benchmarking exercises (Figure 4).

## Tanzania's Milestone Achievement a First for Africa

In December 2018, Tanzania became the first confirmed country in Africa to achieve a well-functioning, regulatory system for medical products. The Tanzania Food and Drug authority made considerable improvements in recent years in ensuring medicines in the healthcare system are of good quality, safe and produce the intended health benefit.

WHO's assessment of the Tanzania Food and Drug authority was based on its Global Benchmarking Tool, which evaluated regulatory functions against a set of 251sub-indicators, such as product authorization, market surveillance and the detection of potential adverse-effects, to establish their level of maturity. One regulatory function namely “NRA lot release” was not assessed during the benchmarking of Tanzanian NRA given the scope of the benchmarking which was limited to medicines and imported vaccines.

The benchmarking of Tanzanian regulatory authorities was carried out in phases by a WHO-led team of international experts. During the first and second quarter of 2018, WHO facilitated self-assessments and conducted a formal evaluation of the Tanzania Food and Drug Authority on the mainland and the Zanzibar Food and Drug Agency and required the regulatory authorities to make a number of adjustments. In the last assessment, Tanzania FDA met all indicators that define a maturity level 3 agency, the second highest on WHO's scale and the target for regulatory systems globally.

## Looking Ahead

The integration of blood and blood products into the GBT has been completed by the end of 2019, and work on integrating medical devices (including diagnostics) into the GBT is still ongoing. Once the integration process is completed, the tool will be known as GBT plus. WHO intends to use Revision VI of the GBT to evaluate and publicly designate national regulatory authorities as WHO-Listed Authorities (WLAs) that have been objectively documented to perform at Maturity Level 3 or 4. A concept note presenting a proposed framework for using the GBT to generate and analyze evidence of regulatory system performance, and to allow for the public listing of regulatory authorities as WLAs, has been made available for public consultation in May 2019. As a result of aforementioned consultation, a policy document was developed and posted for public consultation in December 2019.

## Author Contributions

All authors were involved in developing the concept and methodology as well as drafting and editing the manuscript. All authors developed the figures.

## Conflict of Interest

The authors declare that the research was conducted in the absence of any commercial or financial relationships that could be construed as a potential conflict of interest.
